# Unleashing the potential of women in global surgery: Concrete solutions for achieving gender parity

**DOI:** 10.1371/journal.pgph.0003018

**Published:** 2024-04-04

**Authors:** Rohini Dutta, Carolina Coombes, Anam Ehsan, Mayte Bryce-Alberti, Letícia Nunes Campos, Vanitha Raguveer, Hamaiyal Sana, Mehreen Zaigham, Sabrina Asturias, Shivangi Saha, Kavitha Ranganathan

**Affiliations:** 1 Program in Global Surgery and Social Change, Department of Global Health and Social Medicine, Harvard Medical School, Boston, Massachusetts, United States of America; 2 Mary Horrigan Connor’s Center for Women’s Health and Gender Biology, Brigham and Women’s Hospital, Boston, Massachusetts, United States of America; 3 Faculty of Medicine, Universidad Peruana Cayetano Heredia, Lima, Peru; 4 Department of Plastic Surgery, Brigham and Women’s Hospital, Boston, Massachusetts, United States of America; 5 Faculty of Medical Sciences, Universidade de Pernambuco, Recife, PE, Brazil; 6 Obstetrics and Gynecology Institution of Clinical Sciences Lund, Lund University, Lund, Sweden; 7 Department of Surgery, Universidad Francisco Marroquín, Guatemala City, Guatemala; 8 Department of Plastic Reconstructive and Burns Surgery, All India Institute of Medical Sciences, New Delhi, India; PLOS: Public Library of Science, UNITED STATES

In 1789, Margaret Ann Bulkley, often regarded as the first female surgeon in the United Kingdom, had to disguise herself as a man to practice surgery [1]. Presently, women make up 49% of U.S. surgeons [2], yet global disparities persist, with only 22% and 12.5% female representation among surgeons in Brazil and India respectively [3, 4]. While women no longer need to hide their identities, fostering gender parity in a field entrenched in male achievements remains a universal challenge. Nevertheless, a growing force of women surgeons have successfully defied gender stereotypes and made groundbreaking contributions worldwide.

Women surgeons contribute objective benefits to gender parity. In healthcare, women physicians exhibit higher adherence to clinical guidelines, offer more preventive care, and employ patient-centered communication, leading to improved outcomes and greater patient satisfaction [5]. Organizations with top-quartile gender diversity also showcase better decision-making, effective crisis management, and above-average profits [6].

The global surgery community advocates for safe and affordable surgical care through reform in health policies. The community’s voice can also play a crucial role in addressing and overcoming the challenges hindering gender parity in global surgery. In this piece, we propose concrete solutions to common challenges faced by women surgeons engaging in global surgery work worldwide. While we strongly support equitable parental leave policies, equal pay, and objective promotion criteria, we focus on additional, less-discussed solutions critical for lasting change. These solutions not only empower women surgeons but also promote success across healthcare institutions and organizations internationally.

## Deliberately structure mentorship models that pair female faculty with senior surgeons from diverse backgrounds

Mentorship for women surgeons often relies on other female surgeons, yet it is rarely compensated or academically recognized. The weight of mentorship falls so heavily on women, particularly women of color, that this expenditure has come to be known as the “minority tax” [7]. In addition to developing their own careers within institutions still figuring out how to implement inclusive policies and practices, women are also charged with mentoring the growing next generation of female trainees. While rewarding, this is also time consuming and particularly challenging given that these efforts are not universally recognized.

To address this, establishing mentorship models beyond demographic matching can increasing equal engagement in uncompensated, mission-critical endeavors. This promotes sharing diverse experiences and spreads the time commitment across genders. Tailored partnerships between men in positions of power and young female faculty ensure equal access to opportunities traditionally reserved for "the boys club." Male mentors can support female faculty by including women as co-first authors on collaborative manuscripts, principal investigators on grants, and extending invitations for visiting professorships to deliver lectures. Incentivizing mentoring and implementing flexible work policies fosters equity, helping women surgeons bridge the gender gap in publication metrics and promotion [8]. Similarly, cross-country partnerships can be formalized among male and female surgeons globally, fostering bidirectional learning and inclusivity in the field.

## Creating leadership roles with named titles for early-career female global surgeons

Most surgical leadership positions are held by men. In the US, in 2015 only 3% of department chairs were women, and by 2018, only 18% of fellowship program directors were women–often holding lesser academic ranks than their male counterparts [9]. This persistent underrepresentation is exacerbated by a lack of mentorship, female role models, and gender-biased evaluations [10].

In addition to ongoing efforts to recognize women’s contributions through equitable promotion, there is a crucial need to deliberately create leadership positions for early-career faculty. These roles, with named titles, should extend beyond traditional positions like program directors. Purposeful role creation, diversifying into areas like medical education, mentorship, and community advocacy, can help highlight areas that traditionally go unrecognized. Titles such as director, senior lead officer, and chief can appropriately reflect the prestige of these roles, affirming the status of women. Extending these opportunities to early-career faculty, especially women, has the potential to rectify the pipeline leak issue and emphasize the significance of nurturing the next generation of female leaders in global surgery. This approach not only tackles current imbalances but also sets the stage for a more inclusive and impactful future in the field.

## Develop interventions that address gender-based violence and promote safe working conditions for female colleagues

As advocates for marginalized groups, global surgeons are usually required to travel to a variety of environments domestically and internationally to engage in on-ground research efforts. Unfortunately, this poses safety challenges for women, given the global prevalence of physical and sexual violence affecting one in three women [11]. This alarming statistic has seen little change over the past decade. While male surgeons may often travel alone, it is challenging to ensure the same degree of safety for female surgeons. Risks to women are not limited to low-and-middle countries alone, experts have shared equal risks in affluent Western or developed nations [12]. Therefore, women global surgeons face unique safety concerns, hindering their ability to take advantage of opportunities in novel locations in the absence of proper security measures or funding.

To address this, surgery programs must implement strategies to enhance travel safety for both genders. This commitment to ending gender-based violence includes offering stipends for paired travel, pre-arranging secure transportation, and fostering long-term collaborations that include open discussions on treating female colleagues with respect. Addressing cultural and religious norms and establishing safety monitoring plans before departure is essential for creating a supportive and secure environment for everyone. Increasing the on-ground representation of local women in global surgery research enhances our ability to generate context-specific solutions, promote equity in clinical trial recruitment, and address sensitive health considerations for female patient populations.

## Develop leadership programs to promote evidence-based, collaborative, inclusive governance strategies

The traditional depiction of surgical leadership highlights on paternalistic, commanding, and egocentric ideologies. Data demonstrates that this is ineffective in modern-day practice, leading to a hostile work environment and negatively impacts on learning and patient outcomes [13]. Women leaders like Jacinda Ardern and Tsai Ing-wen showcase effective leadership through collaborative styles and traditionally labeled "feminine" traits such as empathy, humility, and motivation through transformation [14]. Despite this, leadership programs primarily target women, advocating the adoption of traditional leadership practices.

Instead of enforcing gender-stereotypical leadership ideals, we propose a focus on objective criteria supporting positive leadership attributes, aligning with evolving data on effective leadership [14]. While allyship leadership programs have positively influenced dialogue, we suggest more tangible measures for enhanced outcomes. Establishing leadership programs open to men and women emphasizing competence, confidence, "soft skills" for emotional intelligence, and strategies for leading diverse groups benefits society broadly. This approach aims to nurture exemplary role models, shifting away from urging women to conform to traditional male leadership styles. As both men and women depart from conventional gender stereotypes in favor of evidence-based leadership strategies, we pave the way for a societal shift, transcending gender, towards diversity in academia’s leadership.

Women global surgeons have demonstrated their value in healthcare and academia objectively through multiple studies and data sources [5, 6, 15]. To drive progress and promote gender inclusivity, we must address mentorship challenges, designate leadership roles for early-career female surgeons, create diverse leadership positions, intervene against gender-based violence, and promote evidence-based leadership through targeted programs. Securing the future of women global surgeons necessitates implementing innovative strategies that actively support women surgeons and foster inclusivity.
